# Synchrotron X Ray Induced Axonal Transections in the Brain of Rats Assessed by High-Field Diffusion Tensor Imaging Tractography

**DOI:** 10.1371/journal.pone.0088244

**Published:** 2014-02-05

**Authors:** Raphaël Serduc, Audrey Bouchet, Benoît Pouyatos, Luc Renaud, Elke Bräuer-Krisch, Géraldine Le Duc, Jean A. Laissue, Stefan Bartzsch, Nicolas Coquery, Yohan van de Looij

**Affiliations:** 1 INSERM, U836, Grenoble, France; 2 Université Joseph Fourier, Grenoble Institut des Neurosciences, UMR-S836, Grenoble, France; 3 European Synchrotron Radiation Facility, Grenoble, France; 4 University of Bern, Bern, Switzerland; 5 CNRS, CerCo, Toulouse, France; 6 Université de Toulouse, UPS, Centre de Recherche Cerveau et Cognition, Toulouse, France; 7 German Cancer Research Center, Heildeberg, Germany; 8 Service du développement et de la croissance, hôpitaux universitaires de Genève, Genève, Suisse; 9 Laboratoire d'imagerie fonctionnelle et métabolique, école polytechnique fédérale de Lausanne, Lausanne, Suisse; Johns Hopkins Hospital, United States of America

## Abstract

Since approximately two thirds of epileptic patients are non-eligible for surgery, local axonal fiber transections might be of particular interest for them. Micrometer to millimeter wide synchrotron-generated X-ray beamlets produced by spatial fractionation of the main beam could generate such fiber disruptions non-invasively. The aim of this work was to optimize irradiation parameters for the induction of fiber transections in the rat brain white matter by exposure to such beamlets. For this purpose, we irradiated cortex and external capsule of normal rats in the antero-posterior direction with a 4 mm×4 mm array of 25 to 1000 µm wide beamlets and entrance doses of 150 Gy to 500 Gy. Axonal fiber responses were assessed with diffusion tensor imaging and fiber tractography; myelin fibers were examined histopathologically. Our study suggests that high radiation doses (500 Gy) are required to interrupt axons and myelin sheaths. However, a radiation dose of 500 Gy delivered by wide minibeams (1000 µm) induced macroscopic brain damage, depicted by a massive loss of matter in fiber tractography maps. With the same radiation dose, the damage induced by thinner microbeams (50 to 100 µm) was limited to their paths. No macroscopic necrosis was observed in the irradiated target while overt transections of myelin were detected histopathologically. Diffusivity values were found to be significantly reduced. A radiation dose ≤ 500 Gy associated with a beamlet size of < 50 µm did not cause visible transections, neither on diffusion maps nor on sections stained for myelin. We conclude that a peak dose of 500 Gy combined with a microbeam width of 100 µm optimally induced axonal transections in the white matter of the brain.

## Introduction

Microbeam radiation therapy (MRT), an alternative form of brain radiosurgery, is under development since about two decades, and since 15 years [1] at the European Synchrotron Radiation Facility (ESRF) in Grenoble, France. It uses spatially fractionated synchrotron-generated X-rays in the form of arrays of quasi-parallel, lamellar microbeams, tens of micron wide, and spaced hundreds of microns on-center [2]. The quasi-null divergence and the high flux of synchrotron light allows deposition of very high radiation dose (hundreds of Gy) in the brain, ablating selectively neurons, astrocytes and oligodendrocytes in tissue microslices [3]. Normal brain vascular networks appear to be particularly radioresistant to unidirectional spatially fractionated irradiations, as doses up to 1000 Gy do not cause macroscopic necrosis of cerebral tissues [4]–[11]. To our knowledge, no studies dealing with axonal fiber responses to spatially fractionated doses have been reported. Dilmanian *et al.* briefly discussed oligodendrocyte repopulation in the spinal cord 3 months after MRT [12] but the irradiation parameters and biological responses were not extensively described.

Local axonal fiber disruptions might be of particular interest for the management of epilepsy; about two-thirds of the patients who are refractory to antiepileptic drugs are non-eligible for surgery, which is considered to be the therapeutic “gold standard” for epilepsy. Micro- or minibeam irradiation (MBI) can deliver high doses to small and focal targets for the treatment of seizures [13]. However, in mesiotemporal epilepsy, seizures are often generated in large and functional brain regions, a fact that prohibits extensive homogeneous irradiation of such regions. In this work, we aim to perform non-invasive equivalents to common neurosurgical interventions such as corpus callosotomy (CC) and/or multiple subpial transections (MST), using high doses of MBI in the brain of rats. MST is advised for seizures arising from cortical regions that are non-resectable by virtue of their underlying critical neurologic function (such as speech or motor functions). MST, as first described by Morrell *et al.* [14], involves sliding a sharp dissecting instrument subpially within a targeted cortical gyrus, where it severs tangential intracortical fibers, thus disrupting the horizontal spread of an abnormal excitation, while preserving vertical “columnar” connections to maintain function. Corpus callosotomy (CC), a method derived from MST, consists in applying one or two sagittal sections through the corpus callosum to avoid the spread of seizures between the cerebral hemispheres. The sharp dose fall-off of synchrotron light makes exposure to microbeams a credible and non-surgical alternative to both MST and CC. We assume that the high radiation dose deposited in the microbeam paths might act as “x-ray knives”, disrupting neuronal communications. However, the most favorable beamlet width, in-beam (peak) dose and beam spacing that will ensure axonal transections are not defined.

Diffusion Tensor Imaging (DTI) is a Magnetic Resonance technique widely used to map brain architecture by a full description of water diffusion in the tissue. Diffusivity values (mean: MD, parallel: D_//_ and orthogonal: D_⊥_) and fractional anisotropy (FA) derived from DTI are the principal parameters used to assess microstructural brain integrity. A reduction in FA corresponds to a local loss of structural integrity, most of the time associated with a change in diffusivity values. More recently, fiber tractography (FT), ultimate application of DTI which allows a 3D reconstruction of the white matter fibers, has been successfully applied to image diffuse axonal injuries (*i.e.*, axonal fiber transections) following traumatic brain injury [15], [16].

The aim of this work is to assess the effects of MBI in the cerebral white matter of rats by using *ex-vivo* DTI and fiber tractography as well as histopathology. For this purpose, we irradiated the corpus callosum of normal rats in the antero-posterior direction. Several combinations of parameters were tested by changing i) the beamlet width from 25 to 1000 µm, ii) the on center spacing of beamlets from 400 to 2000 µm and iii) the peak dose from 150 to 500 Gy. In order to characterize the effects of these different combinations of MBI parameters on the white matter, we introduced a new metric called irradiated fraction weighted dose (IF_w_D), defined as product of peak dose (PD) multiplied by the beam width (W), then multiplied by the irradiated fraction, i.e., the ratio of beam width to beam spacing (S). Thus, IF_w_D  =  PD x W^2^/S.

## Methods

All operative procedures related to animal care strictly complied with the guidelines of the French Government (licenses 380324/380456 and A3818510002) and were reviewed and approved by the Internal Evaluation Committee for Animal Welfare of the ESRF. The same Committee specifically approved this study. The Anesthesia was induced by isoflurane (5% in air) and maintained by *i.p.* injection of xylazine/ketamine (64.5/5.4 mg.kg^−1^).

### Irradiation: source and parameters

**Figure 1:**
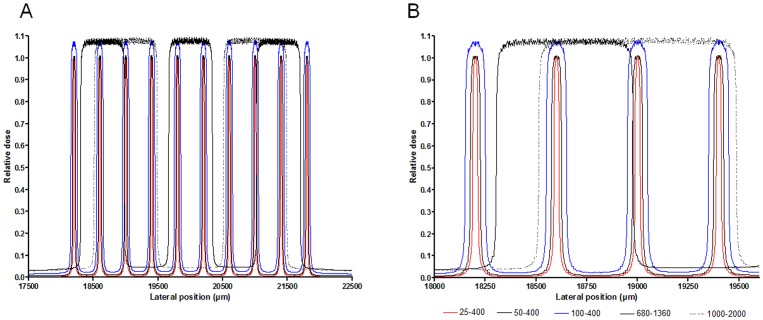
Dose profiles of the different configurations for irradiation. The left panel shows the whole irradiation field while the right panel displays a zoom close to the center of the array of beamlets (25 µm, 50 µm or 100 µm microbeams, all separated by 400 µm on center; 680 µm or 1000 µm wide minibeams, separated by 1360 µm or 2000 µm on center, respectively).

Irradiation with micro- (25 to 100 µm wide) or minibeams (width > 100 µm) (MBI) was performed at the biomedical beamline ID17 of the ESRF. X-rays were produced from a 1.6T wiggler located 43 m in front of the sample. The filtration of the white beam with Be (0.5 mm), C (1.5 mm), Al (1.5 mm) and Cu (1.0 mm) resulted in a spectrum extending from 50 to 350 keV (median energy: 107 keV). The dose rate in air at the surface of the rats was approximately 20,000 Gy.s^−1^. A 4 mm high and 4 mm wide array was defined using tungsten slits. Within this array, quasi-laminar parallel vertical beamlets were produced either by a single scan of the rat through the ESRF multislit collimator [17] for 25–50 µm wide microbeams. To make thicker beamlets, *i.e*., 100 µm, 680 µm and 1000 µm wide, a single slit was used; the beamlets were delivered in a “step and shoot” way, i.e., by repeatedly positioning and exposing rats to a single beamlet to produce the 4 mm×4 mm array. The uniformity and size of the beamlets were checked on radiochromic films and by ionization chamber measurements. The parameters used for irradiation will be noted as beamlet width/on center spacing/dose (µm/µm/Gy) throughout this paper. Anesthetized rats were positioned prone on a computer-controlled goniometer. The upper parts of both cerebral hemispheres were irradiated in the anteroposterior direction as described above. The center of the array was located 4.1 mm below the skull surface and 3 mm laterally of the medio-temporal line. Irradiation parameters and the number of irradiated hemispheres in rat brains are reported in Table 1. Normalized dose profiles are shown in Figure 1.

**Table 1:** Microbeam widths and on center spacings (µm); doses (Gy), numbers of irradiated hemispheres, peak to valley dose ratio and valley doses (Gy).

	Microbeam width (µm)/spacing (µm)					
	25/400	50/400	100/400	680/1360	1000/2000	
**Dose (Gy)**	**Number of irradiated hemispheres**					
150	5	6	6	3	5	
280	4	4	3	3	5	
500	5	4	4	4	3	
	**Peak to valley dose ratio**					
	132.1	93.6	58.2	37.7	40	
**Dose (Gy)**	**Valley dose (Gy)**					
150	1.14	1.6	2.58	3.98	3.75	
280	2.12	2.99	4.81	7.42	7.01	
500	3.78	5.34	8.6	13.26	12.51	

### MONTE CARLO simulations

Dose calculations for the irradiation configurations are based on Monte Carlo simulations. They utilize the source characterization of the ID17 beam line at the ESRF described by Martinez-Rovira *et al.* [18] by incorporating phase space files (PSF). For irradiation fields 1, 2 or 3 cm wide, these phase space files describe energies, positions, directions and the polarization states of photons at a distance of 38.8 m from the wiggler source. In the following, Y denotes the beam axis; the plane of the PSF is at Y = 0. The particles have to be tracked afterwards through a vertical slit of 500 µm width, at Y = 9 cm, and through the multislit collimator (MSC) at Y = 59 cm. The MSC is implemented according to the geometries described in the last paragraph. We have simulated 10^8^ particles leaving the MSC. They enter a three-layered phantom of cubic shape and 5 cm side length. The first layer in the path of the beam, a 1 mm water layer, accounts for the mouse skin. The second is a bone layer of 0.6 mm thickness. We use the ICRU definition of compact bone (web database: http://www.nist.gov/pml/data/star/index.cfm). The third layer is again composed of water and represents soft tissue. The surface of the phantom facing the beam is at Y = 167.42 cm.

Scoring is performed in 5×500×50 µm^3^ (X×Y×Z) bins, where the Z-direction is parallel and the X-direction perpendicular to the microbeams. From these calculations we obtain the dose of a field of 4×0.5 mm^2^ (X×Z) created by the MSC and the vertical slit collimator. To produce a 4×4 mm^2^ field the rat is moved through the beam along the Z axis. The dose for this field is calculated by integrating values along Z from −2 to 2 cm. This results in a dose distribution D(X, Y, Z = 0), *i.e.* a cross section of the absorbed dose in a plane perpendicular to the beams.

### Ex-vivo diffusion tensor imaging and fiber tractography

Two months after exposure the rats were culled. The brains were excised and fixed in PBS/PFA 2% for *ex-vivo* MRI. MR experiments were performed on an actively-shielded 9.4T/31cm magnet (Varian/Magnex) equipped with 12-cm gradient coils (400mT/m, 120 µs) with a transceive 25-mm birdcage volume RF coil. During scanning, the brains were bathed in fomblin (Fomblin Profludropolyether; Ausimont, Thorofare, NJ), an inert oil without MR signal. After manual adjustment of the first and second order shims (waterline width ∼20 to 40 Hz) a Spin-Echo sequence with addition of the Stejskal-Tanner diffusion gradients was used. Diffusion gradients were applied along twelve spatial directions: dual gradient diffusion gradient sampling scheme [19] as well as the six opposite directions to cancel *b*-value cross terms [20]. Intensity, duration and diffusion time were set to 22 G/cm, 3 ms and 20 ms respectively, given a *b*-value of 1185 s.mm^−2^. A field of view of 18×18 mm^2^ was sampled on a 128×128 cartesian grid then zero-filled to 256×256 given an in-plane resolution of 70×70 µm^2^. 20 slices of 0.5 mm thickness were acquired in the axial plane with 10 averages and TE/TR  =  30/2000 ms. Using in house Matlab script (Mathworks, Natick, MA), mean, axial and radial diffusivity values (MD, D_//_ and D_⊥_, respectively) as well as FA were derived from the tensor. The program allows manual delineation of region of interest (ROI) on the FA maps. The external capsule was analyzed at three different levels of the brain (*i.e.* three image planes) and the results were pooled to obtain one data set of DTI derived parameters (*i.e.* MD, D_//_, D_⊥_ and FA) per rat. Significant differences of diffusivity and FA values with the control group were assessed by a Mann-Whitney test using Matlab (Mathworks, Natick, MA). Whole brain fiber tractography was performed using the free software DTI Toolkit (http://trackvis.org/dtk/) with an angle threshold  = 30° and three different FA tracking thresholds of 0.2, 0.3 and 0.4.

Correlations between irradiation parameters and FA values were assessed with Matlab. Linear (Pearson's correlation) and nonlinear (Spearman's correlation) tests were performed.

### Histological procedures

Twenty µm thin coronal brain sections were cut at −20°C on a cryostat (Microm HM560, France) and fixed with PFA 4% for 24 hours. Myelin was stained according to a protocol modified from Gallyas [21]. After incubation in a silver nitrate solution for 1 h, sections were washed in 0.5% acetic acid solution and left in a developer solution freshly prepared as in the original staining procedure, washed in 0.5% acetic acid solution and differentiated in 0.2% potassium ferrocyanide. This step was repeated three times before final fixation in thiosulfate followed by dehydration and mounting.

## Results

### Dosimetry

Figure 1 displays the dose profiles for each irradiation configuration obtained by Monte Carlo simulations. The peak to valley dose ratios and the valley doses delivered for each parameter are given in the Table 1. Depending on the beam size, the PVDRs varied between 37.7 and 132.1; valley doses ranged from 1.1 to 13.3 Gy. Despite of the large range of PVDRs, the effects of the different irradiation configurations were comparable. Indeed, except for the 2 most aggressive irradiation parameters (680 µm/1360 µm and 1000 µm/2000 µm, 500 Gy), valley doses were similar for all the configurations used and remained below 10 Gy.

### Radiation toxicity and survival

**Figure 2:**
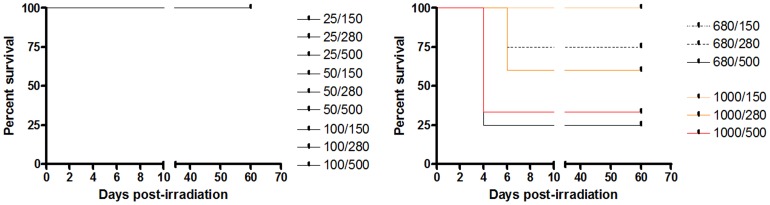
Survival curves of normal rats as a function of the configurations for irradiation. The first number in the legend denotes the width (µm) of the beamlets, the second, the dose (Gy), for instance: 25 µm/150 Gy. All surviving rats were culled at day 60 after exposure.

The figure 2 shows the percentage of rats surviving the different irradiation modes. Only the most aggressive configurations, *i.e.*, (1000 µm/2000 µm and 680 µm/1360 µm) and high radiation doses (280 Gy and 500 Gy) induced animal death within one week after irradiation (survival rates: 33 to 75%). The 1000 µm/2000 µm/500 Gy configuration induced marked hair loss in the paths of the beamlets during the first month following the irradiation. Microbeams (≤100 µm width) did not induce animal death. No neurological changes were observed during the whole experiment (2 months) in rats which survived after the first week; the results did not depend on microbeam width and radiation dose.

### Ex-vivo diffusion tensor imaging

Results are presented as follow: (1) groups showing diffusivity and FA changes, (2) groups showing only diffusivity changes and (3) groups showing no significant modifications when compared to controls.

#### (1) Radiation-induced fractional anisotropy and diffusivity changes

**Figure 3:**
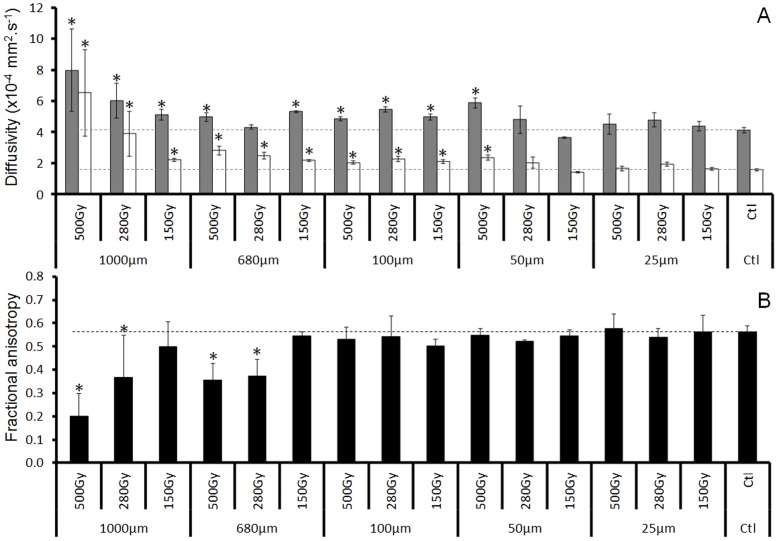
DTI derived parameters in the external capsule. A-Diffusivity values (Axial (grey) and Radial (white) - mean±SEM) as a function of the beamlet width and the dose. “Ctl” denotes sham irradiated control rats. B-Fractional anisotropy values (mean±SD) as a function of beamlet width and dose (*: *P*<0.05, irradiated *vs.* control rat).

Among the 15 groups analyzed, only four groups presented a significant decrease of FA (*P*<0.05) in the external capsule when compared to control values (beam width/on center spacing, µm): 500/1000, 500/680, 280/1000 and 280/680 (Fig. 3A). For each of those beamlet widths, the decrease in FA was related to the dose deposition, *i.e.*, it was much more pronounced at 500 Gy than at 280 Gy (Fig. 3A). These reductions in FA were mainly due to an increase in the D_⊥_, but they were also associated with an increase in the D_//_, resulting in an overall increase of MD (Fig. 3B). Compared to control values, diffusivity values increased in the 4 groups by 41% and 151% for axial and radial diffusivity, respectively. The decrease of FA was −42%.

#### (2) Radiation-induced diffusivity changes only

Compared to control values, diffusivity values (axial and radial) in the external capsule were significantly higher in rats irradiated with the following dose (Gy)/width (µm) configurations: 150/1000, 150/680, 500/100, 280/100, 150/100 and 500/50 (Fig. 3B). FA values tended to be lower than control values, but the difference did not reach significant *P* values (Fig. 3A). Compared to control values, increase in diffusivity values was 28% and 40% for axial and radial diffusivity among the 6 groups. The averaged decrease of FA was −6%.

#### (3) No fractional anisotropy/diffusivity modifications after irradiation

For all the other dose (Gy)/microbeam width (µm) configurations (500/25, 280/50, 280/25, 150/50 and 150/50), no significant differences were observed versus values for FA and diffusivity measured in the control group.

### Fiber tractography

**Figure 4:**
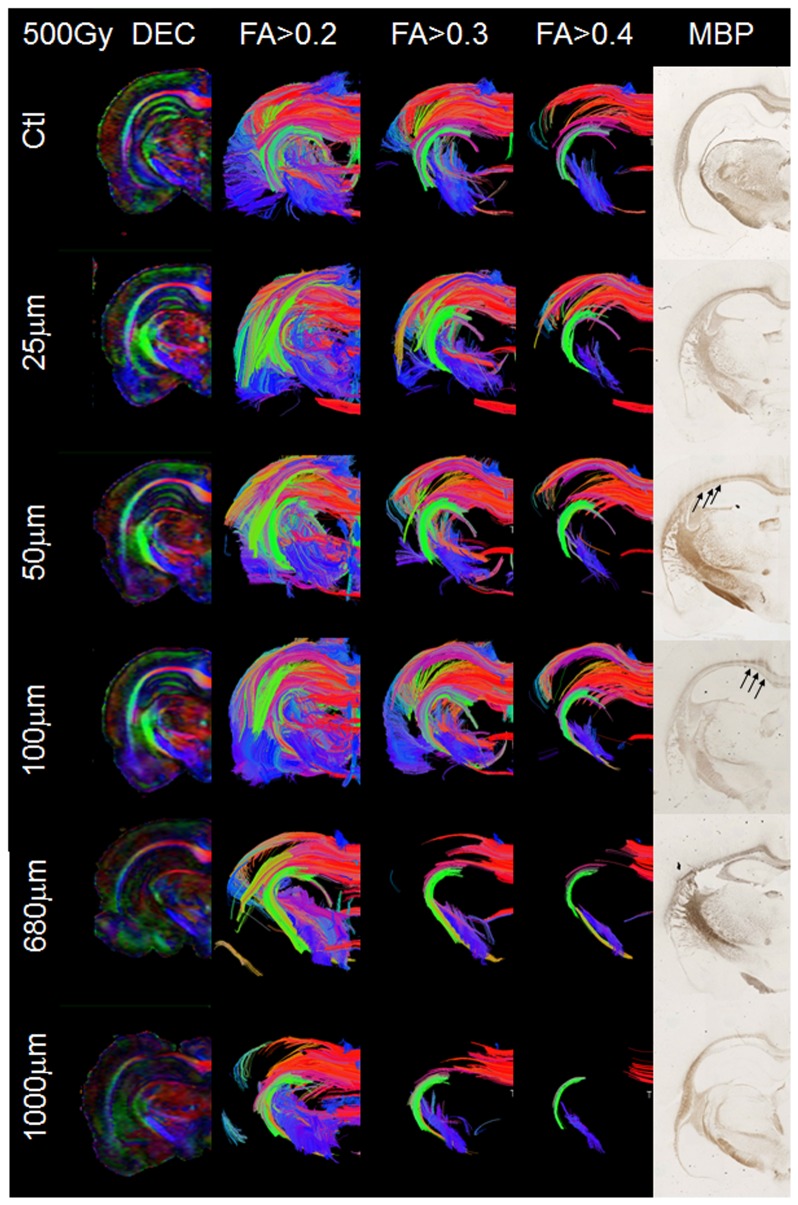
DTI, fiber tractography and histopathology. DTI and MBP stained sections of 5 typical rat hemispheres irradiated with a dose of 500(µm): 1000, 680, 100, 50 and 25. Left column: direction encoded color (DEC) maps; next three columns: 3D fiber tractography images of the irradiated hemisphere computed with FA thresholds of 0.2, 0.3 and 0.4, respectively. Right column: MBP stained coronal section of irradiated hemispheres. Panels in the two bottom rows: Obvious tissue damage after exposure to 1000 µm and 680 µm wide minibeams: white mater loss as depicted by tractography and sections stained for myelin. After exposure to microbeams (25–100 µm) no overt change is observed on tractography maps; conversely, the effects of exposure to 100 µm or 50 µm wide microbeams are visible as pale stripes on sections stained for myelin (black arrows), but not for the 25 µm wide microbeams. Top row: “Ctl” denotes sham irradiated control rats.

The fiber tractography 3D images of the irradiated hemispheres are displayed in figure 4. The reductions of FA in the external capsule are also visible on the direction encoded color (DEC) maps (Fig. 4) with a lower signal for the two wider beamlets (1000 µm and 680 µm wide). No changes were observed on the DEC maps of rats exposed to a higher dose (500 Gy) delivered by narrower beamlets (100 µm, 50 µm and 25 µm wide). Whereas 1000 µm and 680 µm wide beamlets elicited a clear loss of structure detected in the FT images, exposure to minibeams of 100 µm, 50 µm and 25 µm width resulted in densely packed fiber tracts in the external capsule comparable to those seen in control rats.

### Histopathology

The examination of sections of irradiated brains, stained for myelin and shown in figure 4 revealed that damage to the white matter depended more on the beamlet size than on the peak dose. The effects of the 25 µm wide microbeams were not visible in brain sections even after high radiation doses. *A contrario*, external capsule interruptions were detected after exposure to 50 µm or 100 µm wide microbeams. The myelin staining density decreased in each microbeam path (Fig. 4, arrowheads) while it remained comparable to that of control rats between microbeams. No irradiation pattern was visible after exposure to for wider beamlets (680 µm). However, myelin stains could not be made from brains exposed to high radiation doses (500 Gy) and wide beams (1000 µm) because necrotic tissue parts damaged by the irradiation were lost when the frozen sections were made. But even in the brain fragments of the 500 Gy/1000 µm group, it was obvious that the damage was very similar to that seen in rats of the 280 Gy/1000 µm group, with the exception of fragmentation. In the brain of rats exposed to the parameters 500 Gy/1000 µm or 280 Gy/1000 µm, the tissue structure was completely lost in the external capsule; no myelin fibers were detected in the irradiated target zone. Indeed, the path of beamlets could not have been observed because of the extensive demyelination.

### Correlation of DTI and MRT parameters

**Figure 5:**
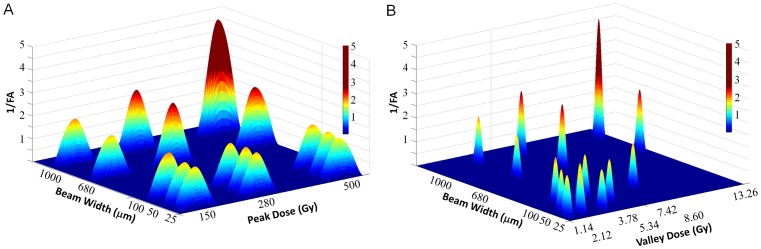
Effects of MRT parameters on white matter structure. A. 1/FA plotted as a function of beamlet width and peak dose. B. Depicts 1/FA versus beamlet width and valley dose. The severity of the damage (low to marked, 1 to 5), depends on the combinations del of beamlet width with peak dose (A) or beam width combined with valley dose (B).

Figure 5 shows the relation between the combined irradiation parameters “beam width/peak dose” as well as “beam width/valley dose” and the white matter damage assessed by the DTI derived parameter 1/FA. Drastic decreases in FA (resulting in intensely red 1/FA peaks in figure 5) were observed after exposure to 1) wide beamlets combined with high peak doses and 2) wide beamlets and high valley doses. Wide beamlets coupled with low peak doses, or wide beamlets coupled with low valley doses did not lead to severe damage as shown by moderate heights of 1/FA peaks. Similar observations were made after irradiations with a high peak dose or a high valley dose when those were combined with narrow beam widths.

**Figure 6:**
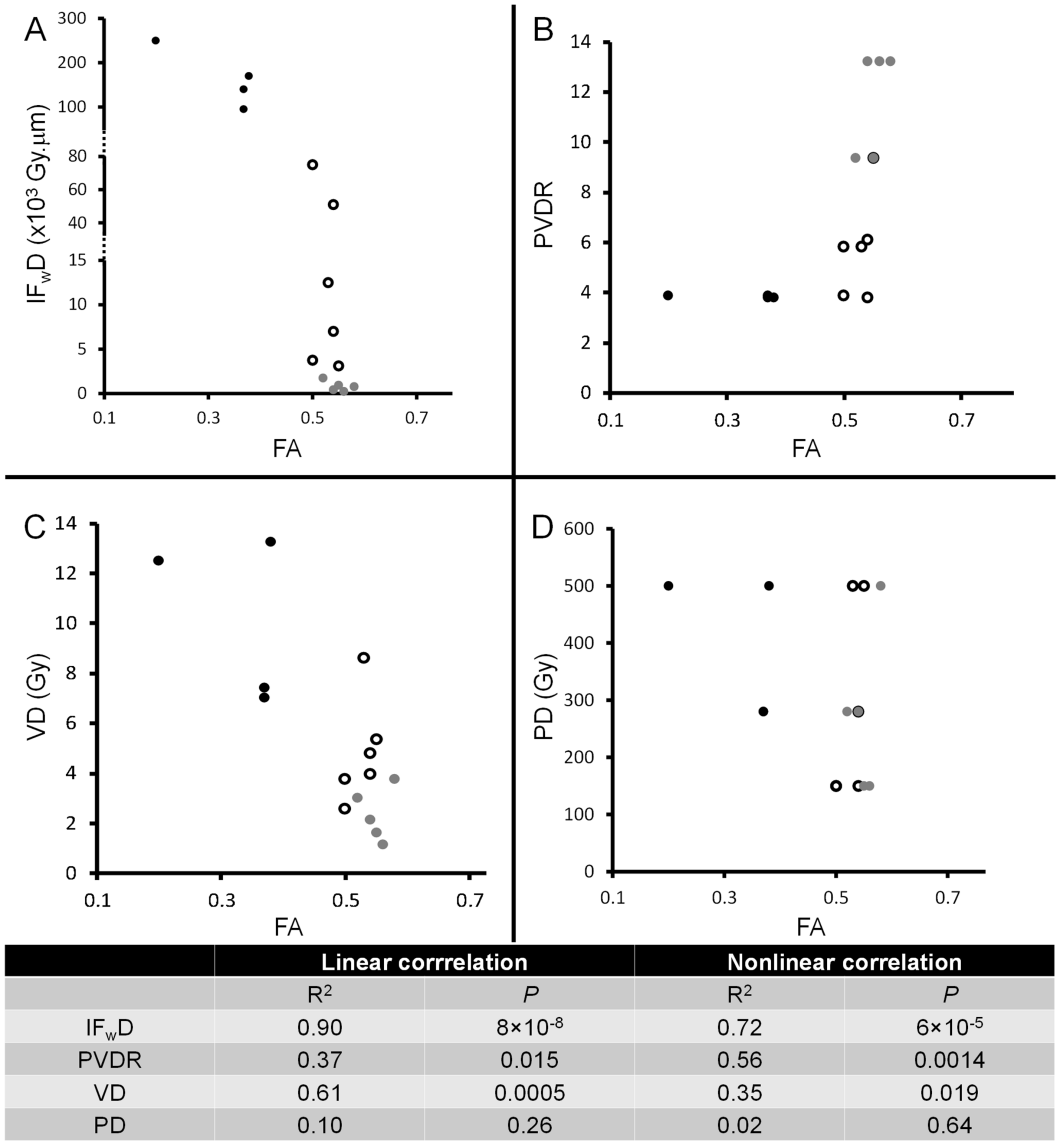
Correlations between MBI parameters for irradiation and FA. A. Irradiated fraction weighed dose (IF_w_D  =  PD×Width× (Width/Spacing)) versus FA. B. Peak to valley dose ratio (PVDR) versus FA. C. Valley dose versus FA. D. Peak dose versus FA. Black disks represent groups showing significant decrease in FA and increased diffusivities; open black circles represent groups showing significantly increased diffusivities with a tendency to FA decreases. Grey disks represent groups without any significant difference compared to controls. Linear (Pearson) and nonlinear (Spearman) correlation coefficients and statistical significance (R^2^ and *P* values) are displayed in the table. Linear and nonlinear correlations were found between FA and MRT parameters.

To assess the influence of MRT parameters on white matter damage several correlations were tested (Fig. 6). We used the metric “irradiated fraction weighted dose” (IF_w_D  =  product of peak dose (PD) multiplied by the beam width (W), then multiplied by the irradiated fraction, defined as ratio of beam width to beam spacing (S). Thus, IF_w_D  =  PD x W^2^/S, expressed in Gy. µ m), was strongly and inversely correlated with the FA values in the external capsule (Fig. 6A), linearly (R^2^ = 0.9, *P* = 8.0×10^−8^) and nonlinearly (R^2^ = 0.72, *P* = 6.0×10^−5^). To illustrate the differences, we plotted the DTI results versus three different markers in figure 6: black disks represent groups showing significantly decreased FA and increased diffusivities; open black circles represent groups that showed significantly increased diffusivities but tended to display decreased FA values. Grey disks represent groups without any significant difference compared to controls. The same notation was used for figure 6B, C and D. A weak but significant correlation was found between peak to valley dose ratio and FA (Fig. 6B), linearly (R^2^ = 0.37, *P* = 0.015) as well as nonlinearly (R^2^ = 0.56, *P* = 0.001). Indeed, valley doses were also correlated with FA values in the external capsule (Fig. 6C), linearly (R^2^ = 0.61, *P* = 0.0005) as well as nonlinearly (R^2^ = 0.35, *P* = 0.019). No correlation was found between peak doses and FA (Fig. 6D).

## Discussion

The aim of this work was to find the optimal irradiation parameters for white matter transections in the rat brain. Responses to several combinations of parameters for irradiation were assessed by DTI and histopathology. The results show that high doses delivered by synchrotron-generated X-rays might be used as a non-surgical alternative to multiple subpial transections or to callosotomy for the treatment of epilepsy. We assume that the appropriate and efficient irradiation parameters are those that do not alter the macroscopic architecture of brain tissues, as characterized by a FA>0.5, while resulting in microscopic demyelination in the microbeam paths. Such results were obtained by using high doses and moderate beamlet widths, *i.e.,* 500 Gy and 100 µm, respectively, and a spacing of 400 µm.

We found a high signal-to-noise ratio in MR images; thus, the diffusion tensor and derived parameters could be determined accurately. However, our diffusion data were acquired *ex vivo,* in fixed brain tissues. Thus, the diffusivity values are significantly lower than in *in vivo* measurements, whereas FA values do not change after fixation [22]. Therefore, altered structures can be detected by way of parameters derived *ex vivo* by DTI in normal and irradiated brains. This method has been used in the past in several animal models [23]–[25].

According to the DTI results, three kinds of effects were observed following irradiation: (1) a drastic FA decrease in the white matter associated with a large increase of the diffusivity values for the 4 most aggressive combinations of high doses (500 Gy and 280 Gy) with wide beamlets (1000 µm and 680 µm); (2) a significant increase of diffusivity values and tendentiously decreasing FA for intermediate irradiation parameters (dose (Gy)/beamlet width (µm): 150/1000, 150/680, 150/100, 280/100, 500/100 and 500/50; (3) no significant difference between irradiated and non-irradiated groups for the lowest irradiation parameters (dose (Gy)/beamlet width (µm): 500/25, 280/50, 280/25, 150/50 and 150/25).

After exposure to wide beamlets combined with high doses, the drastic FA decrease in the irradiated external capsule was accompanied by marked histopathologic alterations. The pattern of injury created by those “highest” irradiation parameters differs from the type of axonal lesions observed after traumatic brain injury or of myelination defects observed in neurodegenerative diseases [16], [26]. Indeed, it has been shown that the decrease of FA in axonal misalignment or in myelination defects is associated with a decreased axial diffusivity [16] or an increased radial diffusivity [26], respectively. This was not the case in the current study. After exposure to the “highest” irradiation mode, even if the increase of D_⊥_ (radial diffusivity) was larger than the increase of D_//_ (axial diffusivity), this freer mobility of water in all directions can be interpreted as a sign of massive macroscopic damage to the white matter in the external capsule. Furthermore, this result is in keeping with the tractography images that show an absence of fibers in the external capsule of the irradiated hemisphere even when the FA threshold was low. Indeed, MBP stained sections reveal an absence of myelin in the irradiated external capsule. Despite different affirmations found in the literature, biological effects of submillimetric “minibeams” have not extensively been described yet [27], [28]. Thus, purported analogies with the normal tissue sparing effect observed after micrometric exposures (“microbeams”) have to be carefully underpinned. Our results indicated that 680 µm and 1000 µm wide minibeams that delivered relatively moderate radiation doses induced complete necrosis of the white matter involving both peak and valley regions, without any distinction. Such combinations of irradiation parameters cause too much collateral damage in addition to the intended local fiber transections. Further, because of the lower PVDR generated by such configurations, only relatively low doses could be delivered when using minibeams, thus reducing the flexibility of the irradiation parameters offered by parallel microscopic exposures: If high peak doses are required to induce efficient and focal white matter transections, they could only be deposited by thin microbeams that warrant a limited valley dose and conservation of macroscopic brain tissue structures.

After exposure to “intermediate” irradiation parameters, microstructural damage was characterized by abnormal diffusivity values despite the absence of significant decrease in FA, although the latter tended to decrease. Thus, the almost normal FA values led to tractography maps that were similar to those of controls, but we detected clear tracks of microbeams on MBP stained sections. The microscopic damage is assumed to be responsible for increasing diffusivity values because it facilitates the mobility of the water. The increase of D_⊥_ (radial diffusivity) was predominant compared to the increase of D_//_. An increase in D_⊥_ can be attributed to local fiber transections that lead to easier diffusion of the water in a perpendicular direction to the fibers in analogy to the abnormal radial diffusivity observed in myelination defects [26]. Irradiation may also reduce barriers of diffusion along the fibers as depicted by increased D_//_ by modifying some elements of the myelin sheath, e.g., oligodendrocytes.

Finally, following exposure to the “lowest” combined irradiation parameters, the white matter structure remained undamaged, perhaps as a manifestation of the well-known radioresistance of the normal brain vascular network [4]–[6], [9], [11] to such microbeams. Indeed, no microbeam paths were seen in MPB stained sections. We suspect that the lesion induced by microbeams has to be large enough to induce death of a certain number of oligodendrocytes in order to generate a visible path in MPB stained sections. Indeed, remyelination might occur within two months after irradiation [12], most likely after lesions induced by exposure to 25 µm wide microbeams.

Elements of oligodendrocytic lineage are considered to be the most proliferating cells in the brain and can be regarded as its most radiosensitive cells [29], [30]. Previous studies have shown that myelin damage after conventional irradiation is reversible up to a certain dose threshold of about 30 Gy [31]. Sun *et al.* recently showed that transplantation of oligodendrocyte precursors cells can reverse functional deficits induced in locomotion of rats 4 months after delivery of 22 Gy (6 MeV X rays) to a 2 cm long segment of the cervical spinal cord [32]. Besides, the current understanding of radionecrosis of the central nervous system also implies a dynamic and continuous response of the glial system. The immediate response of endothelial cells is deemed to lead to an inflammatory cascade that ultimately promotes the delayed effect of irradiation, principally in the white matter [33]. However, reports from Serduc *et al*. have shown that exposure to an “intermediate” combination of parameters for microbeam irradiation, the vascular network is not damaged in normal brain tissue [11]. We can thus assume that our highest irradiation pattern led to irreversible and deleterious damages of both vascular endothelial cells and oligodendrocytes, whereas intermediate combinations led mainly to focal and irreversible damage of oligodendrocytes with no repopulation after exposure. The “lowest” combinations damaged oligodendrocytes focally, but reversibly.

The severity of the damage depends on a combination of parameters, *i.e.,* on beam width/valley dose and beam width/peak dose. As shown in figure 5, the severe injuries with loss of tissue structure were elicited by wide beams associated with high peak or high valley doses. Conversely, a wide beam associated with low peak and/or valley doses did not cause major macrostructural damage, nor did narrow beamlets combined with high valley or peak doses. These results are clearly related to combined parameters with a threshold effect. Whereas ≥ 680 µm wide minibeams associated with a peak dose ≥ 280 Gy and/or a valley dose ≥ 7–8 Gy caused severe macrostructural damage of the white matter, beam widths < 680 µm, peak doses < 280 Gy or valley doses < 7–8 Gy did not damage the white matter as shown by FA and fiber tractography.

For the correlations found between parameters for MBI and FA values in the white matter, it appears that the IF_w_D is strongly correlated with the FA, linearly and nonlinearly. The nonlinearity of the correlation confirms the threshold effect of IF_w_D as depicted by the different markers in figure 6A. The PVDR was also correlated with FA, linearly and nonlinearly, the latter with a larger R^2^. This observation confirms the relevance of the PVDR and the threshold effect linked to this parameter. For the valley dose, the linear correlation is stronger than the nonlinear as revealed by R^2^ and *P* values. This observation confirms the influence of valley dose for the tissue response to MBI. On the other hand, the absence of a correlation between PD and FA shows that the peak dose alone is not important enough to warrant MBI-induced tissue damage. Over the last decades, PVDR was considered as the reference metric for MRT. However, this value does not take into account the beam width, mainly because only thin microbeams (25–50 µm) were used in preclinical experiments. The concept of the “integrated dose” for MRT [34] was defined as the peak dose multiplied by the ratio of the beam width to beam spacing (PD×W/S). Thus the peak dose, weighted with that ratio, reflects also the volume of tissues exposed to the low valley dose. In our study, the new metric IF_w_D reflects the responses of brain tissues to MBI much better because it weights the integrated dose by multiplying it with the square of the beam width. Thus, the IF_w_D displays a steep linear correlation with the beam width, whereas the integrated dose varies only slightly even when beam widths change markedly. Thus, in the current study, two different sets of parameters for beam width, on center spacing and peak dose (680 µm/1360 µm/500 Gy or 1000 µm/2000 µm/500 Gy) induced different tissue responses, but yielded the same integrated dose of 250 Gy. In contrast, the corresponding IF_w_D for those parameters yielded 170,000 gray.µ m or 250,000 gray.µm, respectively, and thus reflected the different postirradiation responses of the brain characterized by different FA values for the white matter (0.38 and 0.20, respectively).

According to our results, IF_w_D values larger than 95×10^3^ Gy.µm induce macroscopic brain damage detectable by a drastic FA decrease on DTI. When the IF_w_D decreases, from 95×10^3^ Gy.µm to 75×10^3^ Gy.µm, a transition was observed, whereas no macroscopic damage occurred after exposure to values smaller than 75×10^3^ Gy.µm. However, some microscopic injuries were detected on MBP stained sections, such as the presence of microbeam tracks. Finally, for values smaller than 1,750 Gy.µm the tissues appeared undamaged after irradiation. Similar observations can be made by calculating IF_w_D values for parameters reported in the literature [3], [5], [11], [35]. Indeed, no histological changes were observed in piglet cerebellum 15 months after a MRT exposure of 263 Gy/25 µm/210 µm (cerebellar peak dose/beam width/spacing), yielding an IF_w_D of 783 Gy.µm [3]. Serduc *et al.* reported no tissue nor vascular damages for an IF_w_D of 975 Gy.µm after exposure to 312 Gy/25 µm/200 µm (peak entrance dose/beam width/spacing) [11]. Laissue *et al.* [35] have described MRT effect in rats bearing intracerebral 9L glioblastoma after a unidirectional hemicranial exposure to 625 Gy/25 µm/100 µm (peak entrance dose/beam width/spacing, respectively) with minor damage in normal brain regions (IF_w_D of 3906 Gy.µm). Our analyses were based on FA values, but retrospective systematic analyses of histological observations published in previous studies, *i.e.* irradiation parameters and MBI-induced tissue changes, would be of particular interest to test the predictive capacity of IF_w_D for MBI-induced brain toxicity.

## Conclusion

The results of the present study show that high dose delivery through synchrotron-generated X-rays might be used as a non-surgical alternative of multiple subpial transections for the treatment of epilepsy. Indeed, our data indicate that quasi non divergent X-ray microbeams can produce highly localized interruptions of the myelin fibers without causing any other histopathologically visible tissue damage in the brain of rats. DTI analyses of the irradiated brains showed that there is a threshold for the preservation of the tissue integrity after irradiation with defined combinations of parameters, i.e., dose and beam size. High radiation doses (500 Gy) interrupted myelin sheaths, but minibeams did not appear suitable for the production of transections because the ensuing brain damage was extensive. Conversely, the damage induced by microbeams (≤100 µm wide) was limited to their paths. No focal necrosis was observed in the irradiated target while overt transections of myelin were detected in histological sections. High radiation doses of 500 Gy, delivered by 50–100 µm wide microbeams spaced 400 µm on center induced localized myelin fiber transections in the cerebral white matter. Finally, we found that a new metric called irradiated fraction weighted dose (IF_w_D) correlated significantly with the shape of altered structures as revealed by DTI derived parameters. Thus, IF_w_D can be used as an accurate marker of effects induced in the cerebral white matter after exposure to arrays of beamlets derived from synchrotron X rays. A peak dose of 500 Gy, delivered by 100 µm wide microbeam spaced 500 µm on center may optimally induce transections of myelin and axons, thus possibly allowing new, clinically relevant applications of MRT.
